# Gut microbiota in PCOS: key microbial changes, potential mechanisms and clinical applications

**DOI:** 10.1186/s12967-026-08352-2

**Published:** 2026-06-05

**Authors:** Shaomin Shi, Jinshu Ma, Xinhui Jing, Jia Wang, Xiaochun Sun

**Affiliations:** 1 Department of Pulmonary and Critical Care Medicine, China-Japan Union Hospital of Jilin University, Changchun, People’s Republic of China; 2 Department of Pathology, China-Japan Union Hospital of Jilin University, Changchun, 130033 People’s Republic of China; 3 Department of Obstetrics and Gynecology, Gongzhuling Central Hospital, Gongzhuling, 136100 People’s Republic of China; 4 Department of Gynecology, China-Japan Union Hospital of Jilin University, Changchun, 130021 People’s Republic of China

## Abstract

**Background:**

Polycystic Ovary Syndrome (PCOS) is a complex endocrine-metabolic disorder manifesting with reproductive and metabolic dysfunction. Its pathogenesis remains incompletely understood, prompting investigation beyond traditional frameworks. The gut microbiota, a key regulator of host metabolism and immunity, has emerged as a significant factor in PCOS development.

**Main body:**

Patients with PCOS exhibit gut microbial dysbiosis, characterized by altered diversity, specific taxonomic shifts (e.g., reduced Lachnospira), and functional impairments including decreased short-chain fatty acid production. This ecological imbalance affects the pathology of PCOS through the “metabolism-immune-endocrine” network, exacerbating insulin resistance, chronic inflammation, and hormonal imbalances like hyperandrogenism, which in turn further disrupts microbial homeostasis. Targeting the microbiota through dietary strategies (e.g., Mediterranean, ketogenic diets), probiotics, prebiotics, and synbiotics shows therapeutic promise in ameliorating PCOS features.

**Conclusions:**

The gut microbiota plays a pivotal role in PCOS pathophysiology and represents a novel therapeutic target. Current limitations include study heterogeneity and unresolved mechanistic details. Future research should focus on large-scale longitudinal studies and developing precise microbiota-based interventions to advance personalized management and improve outcomes in PCOS.

## Introduction

Polycystic Ovary Syndrome (PCOS) ranks among the most prevalent endocrine-metabolic disorders in women of reproductive age globally. Its pathophysiological core lies in the dual dysregulation of reproductive function and metabolic homeostasis, exerting a persistent, lifelong impact on women’s health. During adolescence, PCOS often manifests as menstrual irregularities (e.g., oligomenorrhea, dysfunctional uterine bleeding) shortly after menarche [1]. In reproductive years, it is a leading cause of ovulatory infertility; and in middle to late adulthood, it significantly elevates the risk of long-term complications, including type 2 diabetes mellitus (T2DM), cardiovascular diseases (CVDs), and endometrial carcinoma [2]. This progressive disease burden positions PCOS as a critical public health concern, posing substantial challenges to clinical management and population-level prevention strategies.

Although the clinical hazards of PCOS are well-recognized, its pathogenesis remains a subject of long-standing complexity and multifactorial controversy. Traditional studies have focused on hypothalamic-pituitary-ovary (HPO) axis dysfunction and as core pathways [3], yet they fail to fully explain the disease’s heterogeneity. For instance, why some patients present primarily with metabolic abnormalities, while others exhibit reproductive symptoms as the core feature, and why the efficacy of identical intervention regimens varies markedly across different patients. Recent breakthroughs in microbiomics have offered a new research perspective to address this conundrum. The gut microbiota, regarded as the human “second genome,” not only participates in fundamental physiological processes such as nutrient absorption and immune regulation but also deeply intervenes in the pathophysiological progression of endocrine and metabolic diseases through the complex “microbiota-metabolite-host” crosstalk [4].

Studies have confirmed that PCOS patients commonly exhibit gut microbiota dysbiosis, characterized by decreased α-diversity (with reduced abundance of beneficial bacteria) and altered β-diversity (indicating an imbalanced microbiota structure) [5]. These microbiota perturbations further influence PCOS progression via multiple pathways [6]. Emerging evidence also suggests that dietary interventions can beneficially remodel the gut microbiota, thereby alleviating systemic inflammation, improving insulin sensitivity, and reducing androgen levels to enhance metabolic and reproductive outcomes in PCOS [7]. Tong et al. demonstrated that prebiotic, probiotic, or synbiotic supplementation may exert beneficial effects on multiple health outcomes in women with PCOS [8]. Furthermore, Yanli et al. demonstrated that specific micronutrients and bioactive compounds—including vitamins, minerals, and other agents—can favorably modulate gut microbiota composition, thereby improving PCOS phenotypes [9]. However, current evidence remains largely focused on phenotypic improvements, with relatively limited in-depth analysis of the mechanistic insights through which the gut microbiota regulates PCOS. Based on this evidence, the present review will systematically summarize the association mechanisms between the gut microbiota and PCOS, analyzing from microbiota structural characteristics and metabolic regulatory pathways to clinical intervention evidence. It aims to provide a new dimension for investigating PCOS pathogenesis and offer theoretical support for gut microbiota-based precision diagnosis and treatment strategies.

## The pathophysiological mechanism of PCOS

### The definition and epidemiology of PCOS

According to the 2018 International Evidence-Based Guidelines jointly released by the European Society of Human Reproduction and Embryology (ESHRE) and the American Society for Reproductive Medicine (ASRM), PCOS is defined as a heterogeneous disorder. Its diagnostic criteria are centered on three key features: ovulatory dysfunction (oligoovulation or anovulation), hyperandrogenism (either clinical manifestations or biochemical evidence), and polycystic ovarian morphology. Notably, the diagnosis of PCOS also requires the exclusion of secondary etiologies, including congenital adrenal hyperplasia, Cushing’s syndrome, and androgen-secreting tumors [10].

Overall, PCOS is the most common endocrine disorder affecting women of reproductive age, with a global prevalence ranging from 11% to 13% [11, 12]. Up to 73% of fertility problems are related to PCOS [13]. However, the prevalence of PCOS varies substantially across studies, attributed to differences in diagnostic criteria adopted, the age distribution of study populations, and regional disparities. A study conducted from April to September 2024, sampling 1,201 women aged 10 to 45 years across eight regions of Bangladesh through medical history, physical examination, and blood sample analysis, found the prevalence of PCOS among Bangladeshi women to be 6.9% [14]. In a study of 2,654 American women with a mean age of 35.1 ± 9.6 years diagnosed for PCOS, 2.3% were diagnosed with the condition [15]. This variability in prevalence is likely attributable to differences in diagnostic criteria and regional factors. Regarding the age distribution of study populations, globally, the highest incidence rates of PCOS are observed in the 10 to 14 group and the 15 to 19 group had the highest incidence counts of PCOS, and the incidence of the later age groups declined precipitously [16]. PCOS affects approximately 3% to 11% of adolescents [17]. The prevalence rates of PCOS for Black and White women were 8.0 and 4.8%, respectively [18]. Epidemiological data indicate that overweight or obese women exhibit a higher susceptibility to PCOS [19]. From the perspective of complication epidemiology, women with PCOS face a significantly elevated risk of long-term health complications. Notably, this increased risk of adverse health outcomes-including endometrial cancer and cardiovascular diseases-extends to PCOS patients across all age groups [2, 20].

### Clinical manifestations and diagnostic criteria of PCOS

This disease exerts significant and heterogeneous clinical impacts across the reproductive, metabolic, and psychological domains. Menstrual irregularity constitutes the most common initial symptom of PCOS, presenting as ovulatory dysfunction (oligoovulation) or complete anovulation. A hallmark of PCOS lies in interconnected perturbations of reproductive hormones, with abnormal androgen levels being the most prevalent aberration. Biochemically, this is characterized by elevated serum total testosterone and free testosterone concentrations [21]. Clinical manifestations of hyperandrogenism include hirsutism, acne vulgaris, and androgenetic alopecia. Additionally, women with PCOS exhibit a heightened prevalence of metabolic comorbidities, encompassing insulin resistance (IR), obesity, and dyslipidemia [22]. Psychologically, a subset of patients experiences emotional disturbances. Women with PCOS remain more susceptible to depression and anxiety, and report higher perceived stress levels [23]. Recently, Gao et al. identify four reproducible subtypes-PCOS with hyperandrogen (HA-PCOS), with obesity (OB-PCOS), with high-sex hormone-binding globulin (SHBG-PCOS) and with high-luteinizing hormone–anti-Müllerian hormone (LH-PCOS) through unsupervised clustering of 9 clinical variables in 11,908 affected women, validated across 5 international cohorts which advance understanding of PCOS heterogeneity and provide a framework for subtype-based risk stratification and personalized management [24].

The diagnosis of adult PCOS adheres to the International Evidence-Based Guidelines for the Assessment and Management of PCOS, which mandates meeting two out of the following three criteria [25]: clinical or biochemical hyperandrogenism, ovulatory dysfunction, and/or specific ovarian morphological features (e.g., polycystic ovarian morphology) or elevated anti-Müllerian hormone (AMH) levels. Critically, secondary etiologies must be excluded, such as congenital adrenal hyperplasia, Cushing’s syndrome, androgen-secreting ovarian tumors, and thyroid dysfunction [26]. For adolescent PCOS, diagnosis focuses on two core dimensions: abnormal menstrual cycles and hyperandrogenism. Specific criteria are as follows: ≥2 years after menarche, the presence of menstrual cycles < 21 days (manifesting as dysfunctional uterine bleeding), cycles > 45 days (manifesting as oligomenorrhea), or secondary amenorrhea (amenorrhea duration > 90 days); or primary amenorrhea during the growth and development phase or post-puberty. Concurrently, evidence of hyperandrogenism must be confirmed: clinically, the modified Ferriman–Gallwey score is applied, with a score ≥ 4–6 indicating clinical hyperandrogenism; biochemically, this is defined by elevated testosterone levels or an increased calculated free androgen index (FAI) [27].

### The traditional understanding of the pathogenesis of PCOS

To date, the pathogenesis of PCOS remains incompletely elucidated in clinical practice, with contributions from factors including IR, inflammatory responses, genetics, environment, and gut microbiota imbalance. Among adolescent PCOS patients, 24% have a positive family history of PCOS in first-degree relatives; compared with healthy adolescents, the prevalence of positive family history is significantly higher in adolescent PCOS cases [28]. Within the traditional conceptual framework, the core pathogenesis of PCOS centers on hypothalamic–pituitary–ovarian (HPO) axis dysfunction [29]. Abnormal secretion of gonadotropin-releasing hormone (GnRH) from the hypothalamus triggers excessive secretion of luteinizing hormone (LH) by the pituitary gland, leading to an elevated LH/follicle-stimulating hormone (FSH) ratio [30]. This elevated ratio accelerates testosterone synthesis while inhibiting the proliferation of ovarian granulosa cells and the aromatization of androgens to estrogens, resulting in follicular developmental arrest [31] This manifests as polycystic ovarian morphology and ovulatory disorders. Furthermore, excessive androgen levels exert a feedback effect that exacerbates abnormal GnRH pulsatility in the hypothalamus, forming a pathological vicious cycle. As PCOS is a disease with multiple factors and high heterogeneity, although promising progress has been made in this field over the past few decades, the existing traditional mechanisms cannot explain the pathogenesis of PCOS.

## An overview of the gut microbiota

The human gut microbiome constitutes a diverse and dynamic assemblage comprising bacteria, fungi, viruses, their genetic material, and associated metabolic byproducts, which colonize the human body from birth [32]. At the phylum level, the gut microbiota is predominantly composed of *Bacillota*, *Bacteroidota*, *Actinobacteriota*, *Proteobacteria*, and *Verrucomicrobiota*, with *Bacillota* and *Bacteroidota* collectively accounting for approximately 90% of the total microbial population [33]. However, the relative abundance of these phyla exhibits considerable interindividual variation. Initial microbial colonization is initiated in utero through exposure to maternal microbiota, and is further shaped during delivery [34]. Numerous perinatal and early-life factors—including mode of delivery, prematurity, breastfeeding, host genetics, environmental exposures, maternal infections, obesity, stress, as well as antibiotic and non-antibiotic treatments-significantly influence the assembly of the neonatal microbial community [35, 36]. Thus, while each individual harbors a unique microbial ecosystem, primarily localized within the gastrointestinal tract, these communities share conserved functional traits essential for maintaining host homeostasis and health. In healthy individuals, host-derived factors such as diet, gastric acid secretion, bile acid production, gastrointestinal motility, and immune responses serve as major determinants of microbial composition [37–39]. Furthermore, physicochemical conditions-including intestinal pH, oxygen availability, antimicrobial peptides, and immunoglobulin secretion-govern microbial survival and colonization [40–44].

Often referred to as the “forgotten organ”, the gut microbiota performs multifaceted roles that extend far beyond nutrient digestion, critically contributing to metabolic and immunoregulatory processes. A cardinal function involves the fermentation of complex carbohydrates, such as dietary fibers, to generate short-chain fatty acids (SCFAs)—including acetate, propionate, and butyrate [45, 46]. These metabolites serve not only as the primary energy source for colonocytes and reinforce epithelial barrier integrity, but also enter systemic circulation to modulate immune and metabolic homeostasis [44]. Beyond their nutritional contributions, microbial communities synthesize essential vitamins and facilitate mineral absorption [47, 48]. Additionally, a healthy microbiota provides colonization resistance by producing antimicrobial compounds and competitively excluding pathogens, while SCFAs enhance the mucosal barrier function to prevent translocation of harmful agents. Furthermore, a balanced gut ecosystem confers resistance against pathogen colonization through the secretion of antimicrobial substances and competitive exclusion of harmful microorganisms, whereas SCFAs concurrently strengthen the integrity of the intestinal mucosal barrier to inhibit the translocation of detrimental agents [49, 50]. Notably, the gut represents the largest immune organ in the human body, and the microbiota plays an indispensable role in the development and regulation of host immunity. Microbial metabolites-such as SCFAs, tryptophan-derived indole derivatives, and bacterially modified bile acids (BAs)-orchestrate immune homeostasis by promoting the differentiation of regulatory T cells, curbing excessive inflammation, and fine-tuning immune responses [51–54]. Given this functional complexity, contemporary research has shifted from compositional analyses toward functional characterization of the gut microbiota. Functional redundancy-whereby distinct microbial taxa perform overlapping metabolic roles-and strain-level functional specificity underscore the importance of evaluating microbial functional gene potential and metabolite profiles (e.g., SCFAs and BAs) to elucidate mechanisms underlying health and disease [33]. Such approaches are vital for developing targeted therapeutic interventions. The functional disorder of the gut microbiota has been confirmed to be closely related to the occurrence and development of various systemic diseases through the core mechanisms such as immune, metabolic and barrier disruption. Moreover, it is not limited to the gastrointestinal tract but also affects distant organs through “gut-organ axis” such as the gut-brain axis, gut-liver axis and gut-uterus axis [55–57]. Growing evidence highlights the pivotal role of microbial dysbiosis in the development and progression of endocrine and metabolic diseases, including PCOS [58–61].

## Gut microbiota and PCOS

**Fig. 1:**
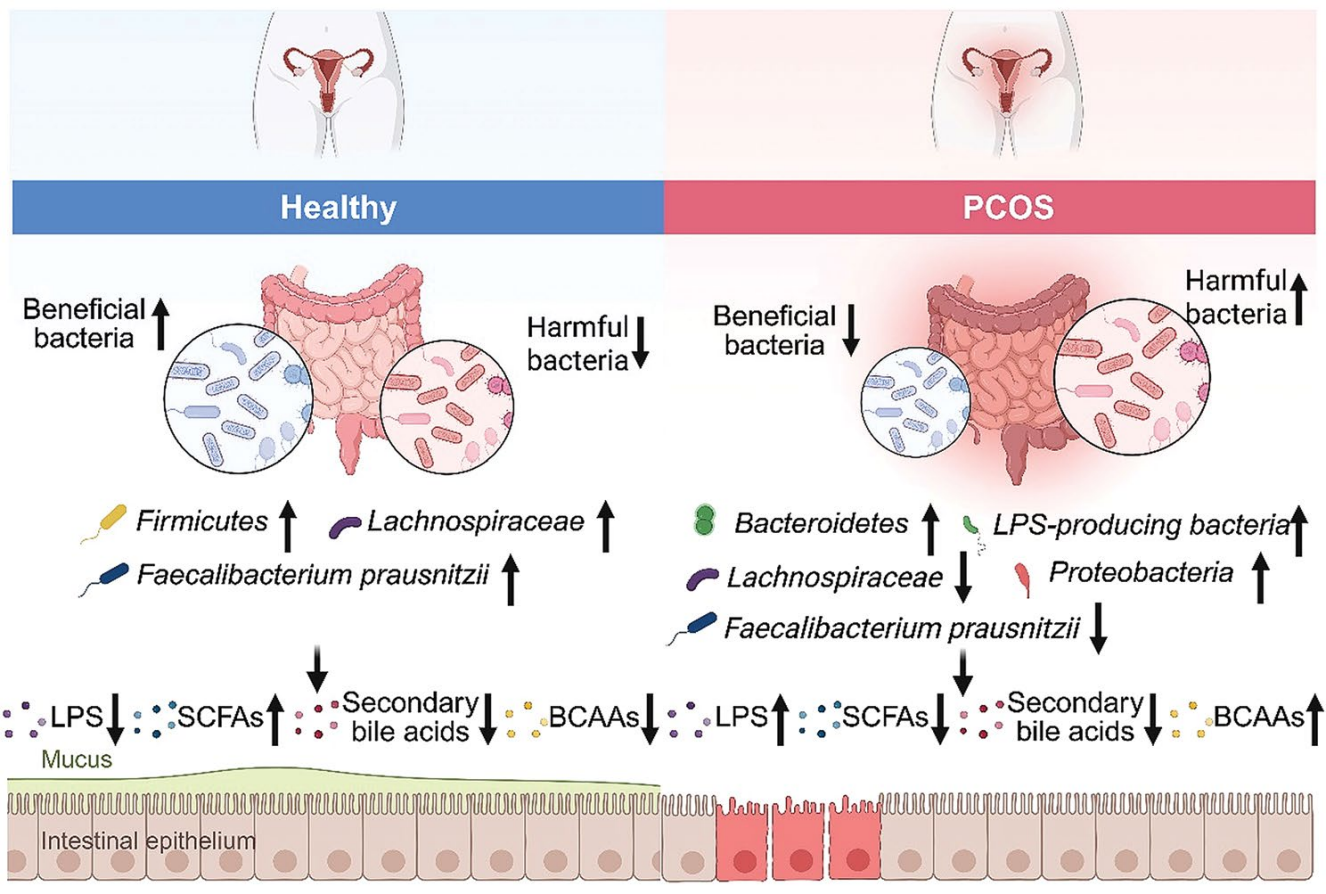
Changes in the gut microbiota in healthy and PCOS population

In 2012, it was first reported that the development of PCOS is associated with gut microbiota dysbiosis [62]. The detailed mechanism is as follows: Gut microbiota imbalance activates the host immune system, triggering chronic inflammatory responses. This, in turn, impairs the function of insulin receptors, leading to a state of IR. The resulting hyperinsulinemia disrupts follicular development. Concurrently, it promotes ovarian follicular cells to overproduce androgens and interferes with normal follicle development. Subsequent research has indicated that the gut microbiota can modulate the pathophysiological alterations of PCOS through its secreted metabolites, while the correlative studies between the gut microbiota and PCOS have been extensively investigated, thus offering novel perspectives for the mechanistic research and intervention strategies of PCOS [63, 64]. Studies based on multi-omics technologies, namely 16S rRNA sequencing, metagenomics, and metabolomics, have preliminarily uncovered the core characteristics of the gut microbiota in PCOS patients. However, there is notable heterogeneity among PCOS patients with different populations and metabolic phenotypes, and some of the findings are still subject to debate (Fig. 1).

### Characteristics of gut microbiota in patients with PCOS

The majority of studies report that the microbiota diversity is reduced in women with PCOS [65, 66]. Research indicates that compared with healthy individuals, the microbial uniformity and phylogenetic diversity of PCOS patients are significantly reduced. Zheng et al. observed that although there is heterogeneity in the relative abundance of bacteria, the depletion of *Lachnospira* and *Prevotella* and the enrichment of *Bacteroides*, *Porphyromonas*, *Lactobacillus*, *Clostridium*, and *Escherichia* in PCOS suggest a shift in the balance of the gut microbiota towards pro-inflammatory bacteria rather than anti-inflammatory ones [61]. Consistently, in the population of obese girls, compared with girls without PCOS, those with PCOS exhibit a lower diversity of the gastrointestinal microbiota, indicating a less healthy state of the microbiota [67]. Notably, these alterations in gut microbiota are not confined to human studies; similar changes have also been observed in animal models of PCOS, suggesting that gut microbiota dysbiosis represents a conserved phenomenon across species in the context of PCOS pathophysiology. Zheng et al. demonstrated that, in a rat model of PCOS, the Chao1 index was significantly decreased compared with the control group, while the Shannon and Simpson indices were also slightly decreased, suggesting lower gut microbial diversity in rats with PCOS [68]. However, Zhang et al. demonstrated that, in a mouse model of PCOS, α-diversity analysis, including the Shannon, Simpson, Chao1, and ACE indices, revealed no significant differences in species richness or microbial diversity compared with the control group [69]. This discrepancy may be partly attributable to the relatively small sample size, which could limit statistical power and obscure subtle alterations in microbial community structure. Nevertheless, although the current evidence remains heterogeneous, most PCOS-related studies, particularly those with relatively larger sample sizes, have reported a reduction in gut microbial diversity. This suggests that decreased microbial richness or evenness may represent a relatively common feature of gut microbiota dysbiosis in PCOS, although this conclusion still requires further validation in larger, multicenter studies with standardized sequencing and analytical approaches.

The changes in the gut microbiota of patients with PCOS are manifested not merely as a decrease in diversity but also as an imbalance in the core bacterial phyla. In general, the fecal abundance of *Actinomycetota* (formerly *Actinobacteria*) and *Proteobacteria* is higher in patients with PCOS than in controls, whereas the abundance of Bacillota (formerly Firmicutes) is lower. Qi et al. found that fecal sequencing of 50 PCOS patients and 43 healthy individuals recruited from Peking University Third Hospital in Beijing, China, showed that compared with the healthy control group, the beta diversity of the microbiome in PCOS patients was significantly reduced, and *Bacteroides vulgaris* showed significant differences between the two populations [70]. Yang et al. found that fecal sequencing of 31 healthy individuals and 56 PCOS patients recruited from Zhujiang Hospital of Southern Medical University in Guangdong Province, China has revealed that in PCOS patients, the phylum Bacteroidota (formerly Bacteroidetes) is significantly enriched while the abundance of the phylum Bacillota is decreased [71]. However, research conducted by Yu et al. revealed that at the phylum level, the abundances of Bacillota, Bacteroidota, Actinomycetota, Tenericutes, and Gemmatimonadetes were all decreased in the PCOS group compared to healthy controls, while the proportions of Proteobacteria, Verrucomicrobia, Fusobacteria, Acidobacteria, and Cyanobacteria were increased in the PCOS group [72]. Upon analyzing the feces of 136 participants, Fu et al. found that the phyla Bacillota and Actinomycetota were abundant in the healthy group, whereas the phyla Bacteroidota and Proteobacteria exhibited relatively lower abundances in the PCOS group [73]. Table 1 provides an overview of gut microbiota dysbiosis in PCOS patients based on evidence from multiple 16S rRNA sequencing studies. Despite these discrepancies across studies, the majority of investigations consistently report a reduction in the abundance of Bacillota in patients with PCOS, suggesting the presence of beneficial bacterial taxa within this phylum that may exert protective effects against PCOS pathogenesis. Further comparison of these studies indicates that *Faecalibacterium*, a beneficial butyrate-producing genus within Bacillota, is frequently depleted in PCOS [76, 77]. Given the reported beneficial effects of butyrate on PCOS, future studies should further investigate whether specific *Faecalibacterium* species or strains exert protective effects against PCOS [78]. By contrast, *Bacteroides* is often enriched in PCOS patients. Since some *Bacteroides* species are considered opportunistic pathobionts and have been linked to inflammatory or infectious diseases, targeting the overrepresentation of *Bacteroides* may represent another potential strategy for microbiota-based intervention in PCOS [79].

**Table 1:** Gut microbiota alterations in PCOS across human and animal studies

Study type	Sample characteristics	α-diversity	Findings (Phyla)	Findings (Genus)	Evidence level	Ref.
Human observational	obesity with PCOS (*n* = 37), obesity without PCOS (*n* = 21)	lower	↑ *Actinomycetota* ↓ *Bacteroidota*	↑ *Prevotella, Finegoldia, Lactobacillus* ↓ *Bacteroides*, *Parabacteroides*	Associative	[65]
Human observational	PCOS (*n* = 66), Control (*n* = 43)	lower		↑ *Porphyromonas spp*, *Bacteroides coprophilus* ↓ *Blautia spp.*, *Roseburia spp.*, *Ruminococcus bromii*	Associative	[66]
Human observational	PCOS (*n* = 1022), Control (*n* = 928)	lower		↑ *Bacteroides*, *Parabacteroides*, *Lactobacillus*, *Fusobacterium*, *Escherichia/Shigella* ↓ *Prevotella*, *Lachnospira*	Associative	[61]
Human observational	PCOS (*n* = 56), Control (*n* = 31)	higher	↑ *Bacteroidota* ↓ *Bacillota*	↑ *Bacteroides* ↓ *Ruminococcaceae*	Associative	[71]
Human observational	PCOS (*n* = 20), Control (*n* = 20)	lower	↑ *Bacteroidota* ↓ *Bacillota*	↑ *Escherichia-Shigella* ↓ *Faecalibacterium*. *Subdoligranulum*, *Prevotella_9*, *Roseburia*	Associative	[72]
Human observational	PCOS (*n* = 98), Control (*n* = 38)	no significant change	↑ *Bacteroidota*, *Proteobacteria* ↓ *Bacillota*, *Actinomycetota*	↑ *Bacteroides* ↓ *Faecalibacterium*. *Prevotella_9*	Associative	[73]
Animal model	PCOS (*n* = 8), Control (*n* = 9)	higher	↑ *Bacteroidota*	↑ *Akkermansia*, *Bacteroides* ↓ *S24-7*	Causal: Human fecal microbiota transplantation	[71]
Animal model	PCOS (*n* = 6), Control (*n* = 6)	lower	↑ *Bacillota*, *Actinomycetota* ↓ *Bacteroidota*	↑ *Ligilactobacillus*, *Lactobacillus*, ↓ *norank__f__Muribaculacea*	Causal: Bacterial supplementation	[74]
Animal model	PCOS (*n* = 5), Control (*n* = 5)	lower	↑ *Bacillota*, *Cyanobacteria*, *Actinomycetota*, *Saccharibacteria*	↑ *Ruminococcus*, *Alistipes* ↓ *Prevotella*	Associative	[68]
Animal model	PCOS (*n* = 8), Control (*n* = 8)	no significant change	↑ *Proteobacteria* ↓ *Bacillota*, *Verrucomicrobiota*	↑ *Roseburia*	Associative	[69]
Animal model	PCOS (*n* = 10), Control (*n* = 10)	lower	↑ *Verrucomicrobiota* ↓ *Bacillota*, *Bacteroidota*	↑ *Akkermansia*, *Bacteroides*, *Turicibacter* ↓ *norank__f__Muribaculacea*, *Lactobacillus*, *Helicobacter*, *Candidatus_Saccharimonas*, *Desulfovibrio*, *Muribaculum*	Associative	[75]

Evidence levels were defined according to the study design. Human observational studies were classified as associative evidence, whereas animal studies involving microbiota manipulation, bacterial supplementation, fecal microbiota transplantation, or microbial metabolite intervention were considered causal evidence

Regarding the inconsistencies observed across different studies, biological, environmental, and methodological factors may collectively explain this heterogeneity. Geographical location, dietary patterns, climatic conditions, and ethnicity vary substantially among study populations and may contribute to inter-individual differences in gut microbiota structure. In addition, BMI and metabolic status are important confounding factors, as obesity, insulin resistance, and chronic low-grade inflammation are commonly associated with PCOS and can independently influence gut microbial composition. However, most existing studies did not stratify participants according to obesity, insulin resistance, or chronic low-grade inflammation, or only considered one of these factors [65]. The intrinsic heterogeneity of PCOS may further contribute to inconsistent findings, as patients with different phenotypes, such as lean PCOS, obesity-associated PCOS, hyperandrogenism-dominant PCOS, or insulin resistance-associated PCOS, may exhibit distinct microbial signatures. Methodological differences may further amplify these discrepancies. Variations in fecal sample collection protocols, storage conditions, processing procedures, DNA extraction methods, amplified 16S rRNA regions, sequencing platforms, reference databases, and bioinformatic pipelines can introduce technical variation, thereby affecting downstream sequencing results and compositional analyses. Therefore, the divergent findings reported in the literature are likely attributable to the combined effects of population characteristics, disease heterogeneity, environmental exposures, and methodological differences. These observations highlight the need for standardized experimental procedures, detailed documentation of clinical and dietary variables, and careful stratification by BMI, metabolic phenotype, ethnicity, and PCOS subtype in future multicenter studies.

Changes in the abundance of gut microbiota alter the microbiota structure, thereby leading to abnormal functional metabolic profiles of the microbiota. These abnormalities include a reduction in the synthesis of SCFAs, an increase in the release of lipopolysaccharide (LPS), and disturbances in bile acid metabolism. Such alterations impact the body’s immune function and the course of PCOS. Hao et al. demonstrated that supplementation with the probiotic strain *Lactiplantibacillus plantarum* NKK20 increased the abundance of SCFAs, particularly butyrate. The underlying mechanism involves SCFA-mediated restoration of intestinal barrier function, thereby reducing LPS levels and attenuating local immune responses within ovarian tissue to alleviate inflammation [80]. This indicates that researchers can reshape the disrupted microbiota structure and modulate the abnormal functional metabolic profiles through prebiotic supplementation, thus potentially ameliorating PCOS. Inulin treatment has been demonstrated to significantly augment the biosynthetic capacity of SCFAs and elevate the levels of SCFAs in feces, thereby mitigating ovarian inflammation in mice with PCOS [81]. Cordyceps sinensis polysaccharides modulate the gut microbiota by decreasing the abundance of Gram-negative bacteria. This action inhibits the production of LPS and its translocation into the systemic circulation. Subsequently, it suppresses the TLR4/MyD88/NF-κB inflammatory pathway in the liver and adipose tissue, restores insulin signaling, and ultimately contributes to the improvement of PCOS [82]. Aberrations in bile acid metabolism can instigate chronic inflammation, exacerbating the metabolic dysfunction of PCOS via multiple mechanisms [6].

### The mechanisms underlying the impact of gut microbiota dysregulation on the PCOS

**Fig. 2:**
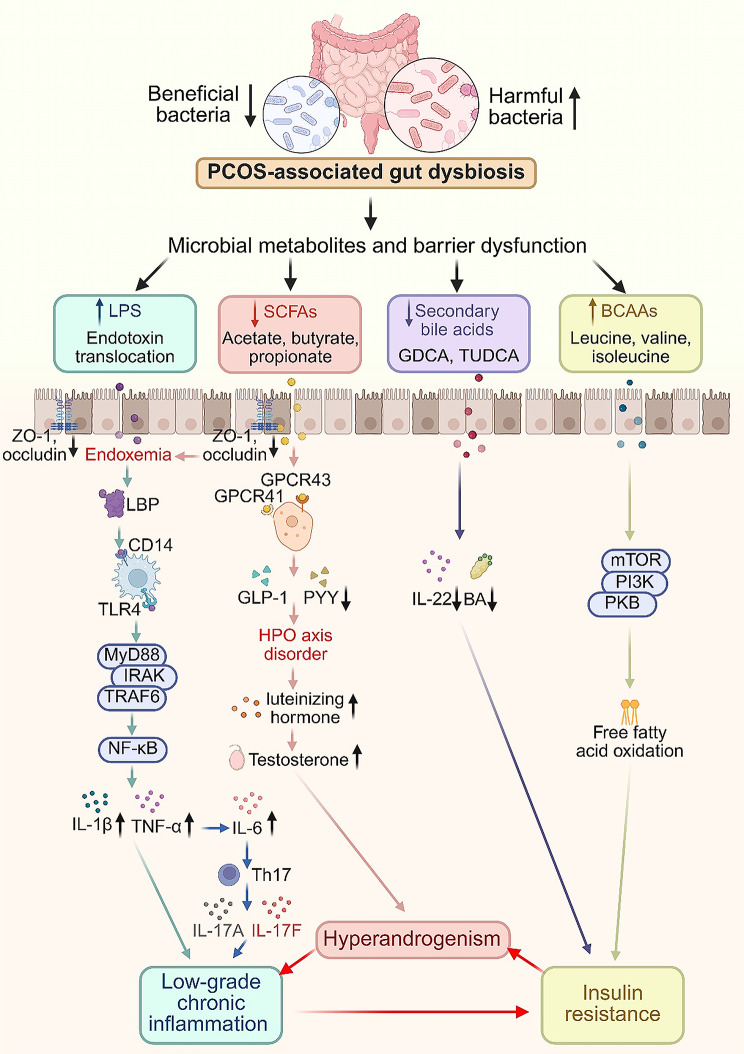
The potential mechanism of gut microbiota in the pathogenesis of PCOS

Accumulating evidence suggests that the gut microbiota, through the interaction of its metabolites with host cell signaling pathways, constructs a metabolism-immunity-endocrine regulatory network across key aspects, including IR, hyperandrogenemia, and chronic inflammation. Moreover, bidirectional feedback regulation exists among these dimensions, and these factors collectively influence the pathological progression of PCOS (Fig. 2).

#### IR and PCOS

IR represents the core pathological characteristic of PCOS. As many as 70% of PCOS patients demonstrate IR [83]. Gut microbiota dysregulation can exacerbate IR. Thus, elucidating the relationship between the gut microbiota and IR is of paramount importance for enhancing the reproductive health of patients with PCOS.

Currently, investigations into the role of IR in the pathological progression of PCOS center on LPS, BAs, branched-chain amino acids (BCAAs), and SCFAs [84]. The gut microbiota acts as a reservoir for LPS. In pathological states, the level of LPS within the intestinal lumen rises, which in turn increases the permeability of the intestinal mucosal barrier, induces intestinal leakage, facilitates the translocation of gut-derived LPS into the bloodstream, and ultimately triggers metabolic endotoxemia. An increase in circulating LPS levels can directly disrupt the function of insulin receptors. Simultaneously, LPS can bind to LPS-binding protein (LBP) and extensively bind to CD14 on T cells, B cells, macrophages, and natural killer cells. Subsequently, LPS is presented to the transmembrane protein TLR4. This interaction activates signaling molecules such as MyD88, interleukin-1 receptor-associated kinase (IRAK), IRAK2, and TNF receptor-associated factor 6 (TRAF6), promoting the production of inflammatory cytokines such as IL-6 and TNF-α. Subsequently, this enhances the IR-promoting ability of LPS through a positive feedback mechanism [85, 86]. In the context of IR in PCOS mice, alterations in the composition of the gut microbiota were observed. Specifically, the abundance of *Akkermansia* was downregulated, and the expression levels of tight-junction proteins, namely Occludin and zonula occludens-1 (ZO-1), decreased. Concurrently, the level of LPS increased. These findings suggest that gut microbiota dysbiosis can trigger metabolic endotoxemia, exacerbate the systemic inflammatory response, and contribute to the development of IR in patients with PCOS [75]. Bacterial species within the gut microbiota, including *Lactobacillus*, *Bifidobacterium*, and *Listeria*, are able to transform primary BAs into secondary BAs via dehydroxylation reactions. Perturbations in the gut microbiota can result in a decrease in BAs activation, thereby inhibiting glucose metabolism and insulin sensitivity [87]. Studies have shown that the feces of patients with PCOS can induce ovarian cysts and IR in mice. The relatively high bile salt deconjugation ability in these feces causes a notable decrease in the levels of conjugated secondary BAs, namely glycodeoxycholic acid (GDCA) and tauroursodeoxycholic acid (TUDCA). Exogenous supplementation of GDCA and TUDCA can improve the PCOS phenotype in mice by modulating the BA/IL-22 signaling axis [70]. Furthermore, when BAs production is compromised, the overgrowth of small intestinal bacteria and augmented intestinal permeability can allow toxic substances to enter the systemic circulation. This phenomenon gives rise to endotoxemia and induces IR, suggesting that dysbiosis and BAs imbalance reinforce each other [85]. Recent research has indicated that individuals with IR exhibit elevated levels of BCAAs in their serum metabolome. Furthermore, this alteration has been associated with an increase in the abundance of *Prevotella copri* and *Bacteroides uniformis*. Notably, *P. copri* has the potential to induce IR, exacerbate glucose intolerance, and elevate BCAA levels [88]. Zhang et al. conducted an analysis involving 111 women in Beijing, China, which revealed that the IR-PCOS group exhibited significantly elevated levels of BCAAs, including leucine, valine, and glutamic acid, when compared to the non-IR PCOS group. Moreover, high levels of BCAAs were also correlated with a lower pregnancy rate and a higher miscarriage rate [89]. Similar trends have also been observed in animal research, the supplementation of BCAAs in the diet of mice elevated the risk of developing IR [90]. Gut microbiota dysbiosis is a crucial factor underlying this relationship. In patients with PCOS, gut microbiota dysbiosis is manifested as an elevated abundance of genera capable of producing BCAAs, such as *Streptococcus* and *Prevotella*, which produce more BCAA [65, 91]. BCAAs can enhance the oxidation of free fatty acids, trigger the activation of the mTOR/PI3K/PKB pathway, and consequently induce IR [92]. In patients with PCOS, gut microbiota dysbiosis is associated with reduced levels of SCFAs. SCFAs interact with GPCR43 and GPCR41 in enteroendocrine cells, intestinal epithelial cells, and pancreatic β-cells. This interaction leads to an increased secretion of glucagon-like peptide 1 (GLP-1) and peptide YY (PYY), which in turn regulates the HPO axis [93]. Moreover, it modulates the levels of LH and testosterone, thus exerting an impact on the progression and phenotypic manifestations of PCOS [94]. Numerous studies have demonstrated that the abundance of SCFAs-producing bacteria, particularly butyrate-producing bacteria, is decreased in individuals with IR. Enhancing the abundance of these bacteria can mitigate inflammation and disorders in IR individuals [95, 96]. This finding suggests that SCFAs not only play a role in influencing the hormonal imbalance associated with PCOS, but are also of critical importance in the development of IR-PCOS.

#### Hyperandrogenism and PCOS

Hyperandrogenism, one of the clinical characteristics of reproductive abnormalities in PCOS, has been associated with the crucial role played by the gut microbiota in the pathophysiological progression of this disorder [97, 98]. Among women diagnosed with PCOS, an elevation in LH prompts the ovarian theca cells to generate an excessive number of androgens [99]. Testosterone, the most pivotal member among androgens, has the potential to impact the composition of the female gut microbiota. Studies have indicated that there exists a negative correlation between the α-diversity of the gut microbiota and the overall testosterone levels in the body, thereby demonstrating the association between the two [66]. Beyond these associative findings in humans, animal models have provided causal evidence that alterations in gut microbiota diversity can directly influence circulating testosterone concentrations. The depletion of the microbiota results in an elevation of circulating testosterone concentrations in female mice whereas it brings about a reduction in male mice, which indicates a reciprocal relationship between the quantity of androgen and the microbiota [100]. The causal relationship between the gut microbiota and androgens remains a matter of great contention in current research; the majority of studies postulate that elevated androgen levels bring about alterations in the composition of the gut microbiota.

The ovariectomized and DHT-treated mice exhibited metabolic phenotypes resembling PCOS, including hyperandrogenemia and IR. Notably, alterations in the gut microbiota were observed in these mice, with significant changes noted in taxa such as *S24-7*, *Rikenellaceae*, and *M. schaedleri*; specifically, the abundance of *S24-7* was significantly elevated in the OVX+DHT group [101]. In a Trazole-induced hyperandrogenic mouse model of PCOS, the diversity of the gut microbiota was found to be reduced. Moreover, it was further revealed that hyperandrogenemia could induce IR and metabolic abnormalities in PCOS by modifying the structure of the gut microbiota [102]. Regression analysis demonstrated that the reduced abundance of several genera in mice with PCOS was correlated with elevated circulating testosterone levels [103]. A number of studies postulate that alterations in the gut microbiota give rise to aberrations in androgen metabolism. Recent investigations have advanced the gut microbiota-testis axis hypothesis, indicating that the gut microbiota serves as a key regulator of androgen production and metabolism. Moreover, it has the potential to penetrate the blood-testis barrier (BTB) to modulate testicular function and exert an impact on spermatogenesis [104].

#### Low-grade chronic inflammation and PCOS

Numerous investigations have documented that individuals with PCOS manifest a chronic inflammatory state [105, 106]. In the ovarian tissues of PCOS patients, a substantial infiltration of macrophages can be detected, and macrophages are among the cell types that participate in inflammatory regulation [107]. In patients with PCOS, inflammatory markers exhibit varying degrees of elevation, including TNF-α, C-reactive protein (CRP), IL–8, and IL-6 [108]. These macrophages secrete inflammatory cytokines, which can lead to local and systemic chronic inflammation in PCOS [3]. The majority of investigations regarding the chronic low-grade inflammatory state in PCOS have centered on the measurement of CRP, an acute-phase protein that is generated subsequent to stimulation by inflammatory cytokines. Kelly et al. initially compared 17 females with PCOS and 15 healthy controls. The findings indicated that the concentration of CRP in females with PCOS was significantly increased (geometric means: 2.12 and 0.67 mg/L, respectively; *p* = 0.016) [109]. A substantial number of subsequent studies have further corroborated that PCOS can give rise to an elevation in CRP [110, 111]. The elevation of circulating IL-6 levels triggers the activation of the pro-inflammatory Th17 cell subset. Th17 cells, by increasing the expression of IL-21, stimulate the release of the inflammatory cytokines IL-17A and IL-17F. Concurrently, TNF-α not only directly stimulates the expression of IL-6 but also synergizes with IL-23 to enhance the expression of IL-17A. This process leads to inflammation - associated oxidative stress and thereby contributes to the development of the inflammatory profile in PCOS [29]. One of the underlying origins of chronic inflammation lies in the gut microbiota within the intestinal tract that possess the ability to produce LPS. Upon the disruption of bacterial homeostasis, a greater quantity of LPS is secreted, infiltrating the organism and potentially gaining entry into the bloodstream [112]. In the presence of LBP, CD14, and myeloid differentiation factor 2 (MD-2), LPS is recognized and bound by TLR4, thereby giving rise to a wide range of chronic inflammatory disorders [113]. Findings consistent with this research indicate that within the intestinal tract of patients with PCOS, certain Gram-negative bacteria belonging to the genera *Bacteroides* and *Escherichia/Shigella* exhibit a notable increase [114]. At the same time, certain medications can reduce LPS production and improve the phenotype of PCOS by reshaping the homeostasis of gut microbiota. Nevertheless, direct clinical evidence investigating this therapeutic mechanism in humans remains limited. This notion has been substantiated primarily through animal models, which provide critical mechanistic insights. Wang et al. found that the Bu Shen Hua Zhuo formula (BSHZF) is associated with an increase in α diversity of gut microbiota, a decrease in relative abundance, and an enhancement in SCFAs-producing bacteria. Furthermore, BSHZF exhibited a negative correlation with serum LPS levels and TLR4 expression, inhibiting the activation of NF-κB signaling-mediated inflammatory responses related to PCOS within ovarian tissues [115].

### Confounding factors and causal interpretation in PCOS microbiome studies

Although gut microbiota alterations have been widely reported in patients with PCOS, these findings should be interpreted with consideration of potential confounding factors, including obesity, insulin resistance, dietary patterns, and medication use. These factors can independently shape gut microbial composition and may partly contribute to the differences observed across studies. Nevertheless, they do not weaken the biological significance of the gut microbiota. Instead, they highlight the gut microbiota as a potential integrative mediator linking host metabolic status, dietary exposure, therapeutic intervention, and endocrine dysfunction to PCOS pathophysiology.

Obesity and insulin resistance are important factors that can shape gut microbiota composition in PCOS. Since many patients with PCOS are overweight or insulin resistant, some microbial changes observed in PCOS may partly reflect obesity- or metabolism-related alterations. However, this does not weaken the potential functional role of the gut microbiota. Rather, obesity or metabolism-associated alterations may reshape the gut microbiota, which may subsequently influence PCOS-related metabolic features through the regulation of short-chain fatty acid production, intestinal barrier function, low-grade inflammation, and bile acid metabolism [116]. Thus, obesity and insulin resistance should be considered not only as confounders, but also as metabolic contexts in which gut microbiota-mediated mechanisms may participate in PCOS progression. Dietary patterns represent another major determinant of gut microbiota structure and function. Variations in total energy intake, dietary fiber consumption, fat intake, carbohydrate quality, and regional dietary habits can markedly influence microbial diversity and the abundance of beneficial metabolite-producing bacteria. These diet-induced microbial changes may further affect inflammatory and metabolic pathways relevant to PCOS [63]. Similarly, metformin, which is widely used to improve insulin resistance in PCOS, can substantially reshape the gut microbiota and microbial metabolism [117]. This suggests that at least part of the therapeutic effect of metformin may be mediated through gut microbiota modulation, further supporting the functional importance of the microbiome in PCOS. Hormonal treatments, including oral contraceptives, progesterone, and anti-androgen therapies, may also influence gut microbial communities by altering endocrine status, bile acid metabolism, immune responses, and host metabolism.

Taken together, these observations indicate that PCOS-related gut microbiota changes should be interpreted within the broader context of host metabolism, diet, medication exposure, and endocrine status. Although these factors may confound the identification of PCOS-specific microbial signatures, they also reinforce the concept that the gut microbiota is an active component of the metabolic and inflammatory network involved in PCOS. Therefore, future studies should avoid attributing microbial alterations solely to PCOS.

## Gut microbiota-based intervention strategies for PCOS

**Fig. 3:**
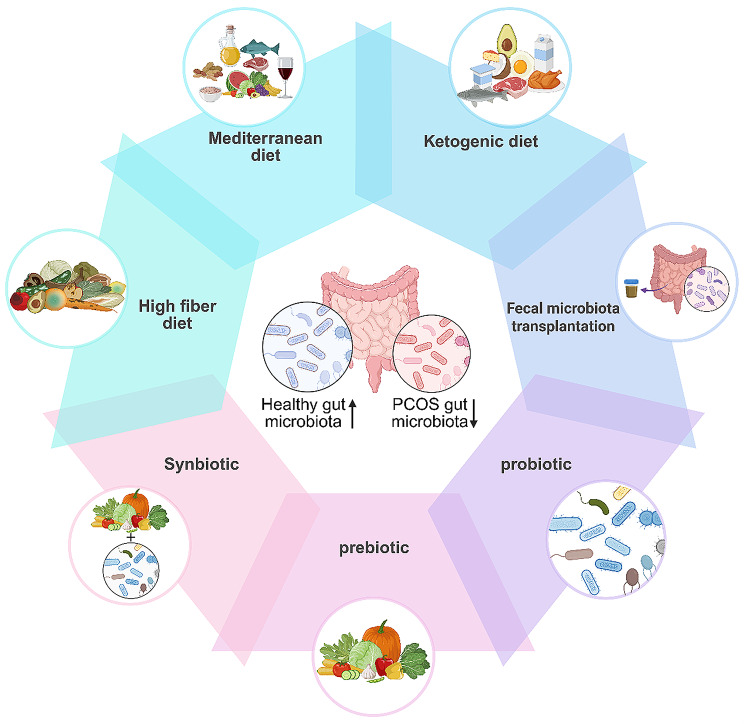
Microbiome-based strategies for PCOS

Alterations in the composition and function of the gut microbiota play a significant role in either accelerating or alleviating the pathophysiological progression of PCOS. Investigating the gut microbial ecosystem is essential for identifying novel therapeutic targets and treatment strategies for PCOS. Accumulating evidence suggests that targeting the gut microbiota may offer promising approaches for the prevention and management of PCOS. Several interventions-including dietary modifications, probiotics, prebiotics, and FMT-have been employed to correct dysbiosis, restore microbial homeostasis, and improve clinical outcomes in PCOS and other reproductive disorders. Table 2 presents a summary of recent dietary, probiotic, prebiotic, and synbiotic intervention strategies for PCOS (Fig. 3).

**Table 2:** PCOS intervention strategies based on diet, probiotics, prebiotics, and synbiotics

Therapeutic strategy	Species	sample characteristics	Experimental Cycle	Results	Ref.
Diet	Mediterranean Diet	PCOS (*n* = 112), Control (*n* = 112)	7 days	Inverse correlation between Mediterranean diet adherence and PCOS severity.	[118]
Diet	Mediterranean diet combined with a low-carbohydrate dietary model (MED/LC) or the Low fat diet (LF)	MED/LC (*n* = 36); LF (*n* = 36)	12 weeks	MED/LC diet model is a good treatment for overweight PCOS patients, significantly restoring their menstrual cycle, improving their anthropometric parameters and correcting their disturbed endocrine levels, and its overall effectiveness is significantly better than the LF diet model.	[119]
Diet	Reduce sweets, fatty red meat, and alcohol intake	PCOS (*n* = 154)	6 months	Women who underwent dietary modification demonstrated significantly lower levels of total testosterone, androgens, and body mass index compared to those without such intervention.	[120]
Diet	Ultray low-calorie diet (VLCD) or moderate energy deficit diet	VLCD (*n* = 21); moderate energy deficit diet (*n* = 19)	8 weeks	The VLCD resulted in significantly greater weight reduction and was accompanied by more pronounced improvements in hyperandrogenaemia, body composition, and several metabolic parameters in obese women with PCOS as compared to the energy deficit approach.	[121]
Diet	Ketogenic diets or very-low-energy ketogenic therapy	10 studies	Multiple cycles	The ketogenic diet and the very low-calorie ketogenic diet both significantly improved the PCOS phenotype.	[122]
Diet	Ketogenic diets	PCOS (*n* = 14)	12 weeks	Body mass index, fat mass, visceral adipose tissue, triglycerides, total cholesterol, and low-density lipoprotein cholesterol were significantly reduced, along with an increase in high-density lipoprotein levels.	[123]
Diet	Ketogenic diet or modestly hypocaloric Mediterranean diet	ketogenic diet (*n* = 73); modestly hypocaloric Mediterranean diet (*n* = 71)	45 days	Both dietary methods improved the phenotype of PCOS patients.	[124]
Diet	Portfolio moderate-carbohydrate (PMCD) or ketogenic diets	PMCD (*n* = 21); ketogenic diets (*n* = 19)	8 weeks	Both diets showed an increase in body measurement indices, metabolic status, and reproductive hormone levels.	[125]
Diet	Ketogenic diets	PCOS (*n* = 30)	3 months	All women (*n* = 30) had restoration of regular menstrual cycles.	[126]
Diet	Ketogenic diets	PCOS (*n* = 18)	45 days	Gradual carbohydrate reintroduction following ketogenic diet intervention successfully restored menstrual cyclicity in women with PCOS and oligomenorrhea.	[127]
Diet	Ketogenic diets (β-hydroxybutyrate)	PCOS (*n* = 20)	10 hours	Ketone supplementation acutely lowers androgen and glucose levels in women with PCOS.	[128]
Diet	high fiber or low glycemic index (LGI)/low glycemic load (LGL) dietary	13 studies	Multiple cycles	Optimizing the quality of dietary carbohydrates has significant benefits for the glucose and lipid metabolism, sex hormone levels, and weight of women with PCOS.	[129]
Diet	High-protein hypocaloric diet or calorie-restricted diet	PCOS (*n* = 72)	3 months	HThe high-protein hypocaloric diet group performed exceptionally well in maintaining the fat-free mass and fat-free mass index of patients with PCOS, and the reduction in body fat percentage was more significant.	[130]
Diet	High-fiber diet or combined with acarbose	High-fiber diet (*n* = 14); High-fiber diet combined with acarbos (*n* = 11)	12 weeks	High-fiber diet could alleviate the chronic metabolic inflammation, reproductive function, and brain–gut peptides secretion of patients with PCOS, and high-fiber diet combined with acarbose could better improve the PCOS clinical phenotypes.	[131]
Diet	dulaglutide and calorie-restricted diet (CRD)	dulaglutide combined with CRD (*n* = 35); CRD (*n* = 33)	6 months	Compared with the treatment with CRD alone, dulaglutide combined with CRD shows a more significant effect in improving PCOS.	[132]
Probiotic	Familact probiotic capsule (500 mg)	Probiotic supplements (*n* = 36); placebo (*n* = 36)	8 weeks	After probiotic treatment, the serum insulin level significantly decreased.	[133]
Synbiotic	*Lactobacillus*, *Bifidobacterium Streptococcus*, and inulin-type prebiotics (Fructooligosaccharides)	Synbiotic supplements (*n* = 34); placebo (*n* = 34)	8 weeks	Synbiotic supplementation improved glycemic indices, lipid profile and obesity values in women with PCOS.	[134]
Prebiotics	Resistant dextrin	Prebiotics supplements (*n* = 31); placebo (*n* = 31)	3 months	Resistant dextrin consumption can regulate metabolic parameters and androgen levels and manifestations including hirsutism and menstrual cycle irregularity in women with PCOS.	[135]
Synbiotic	pomegranate juice, synbiotic beverage or synbiotic pomegranate juice	pomegranate juice (*n* = 23), synbiotic beverage (*n* = 23) or synbiotic pomegranate juice (*n* = 23)	8 weeks	The insulin sensitivity in both the synbiotic treatment groups significantly improved, and the testosterone levels also decreased.	[136]
Synbiotic	*Lactobacillus*, *Bifidobacterium*, *Streptococcus*, and inulin	Synbiotic supplements (*n* = 44); placebo (*n* = 44)	12 weeks	Synbiotic partially improved the clinical symptoms of patients with PCOS.	[137]
Synbiotic	*Lactobacillus*, *Bifidobacterium* and inulin	Synbiotic supplements (*n* = 30); placebo (*n* = 30)	12 weeks	Synbiotic significantly improves IR, triglyceride levels, VLDL-cholesterol concentrations and AIP biomarker levels in women with PCOS.	[138]
Synbiotic	*Lactobacillus*, *Lacticaseibacillus*, *Bifidobacterium*, *Streptococcus*, and 21 mg inulin	Synbiotic supplements (*n* = 47); placebo (*n* = 47)	12 weeks	Synbiotic supplementation in PCOS women for 12 weeks had a beneficial effect on sex hormone-binding globulin but did not affect other clinical and hormonal parameters.	[139]

### Diet intervention

Dietary intervention represents one of the most direct and effective natural strategies for modulating the structure and function of the gut microbiota. Specific dietary patterns can not only ameliorate metabolic and endocrine parameters in women with PCOS but also confer long-term benefits by promoting a healthier microbial ecosystem. Epidemiological studies reveal significant geographic variations in the prevalence of PCOS. East Asian regions generally demonstrate lower prevalence rates (5.6%-6.3%), while South Asian populations exhibit remarkably high rates of polycystic ovarian morphology (52%) [140]. Among Hispanic women, particularly Mexican-Americans in the United States, the prevalence is significantly elevated (approximately 13%) with more severe metabolic manifestations compared to other ethnic groups. Indigenous populations undergoing rapid socioeconomic transitions, such as Australian Aboriginal women, show exceptionally high prevalence rates (up to 26%) [140]. A critical question arises whether these disparities are associated with distinct lifestyle and dietary patterns across regions. Research indicates that dietary habits may serve as important contributing factors: East Asian populations typically consume diets high in refined carbohydrates with moderate vegetable intake, whereas populations experiencing rapid transition toward Western dietary patterns characterized by high saturated fats, sugars, and processed foods demonstrate substantially elevated PCOS incidence, suggesting these dietary shifts may represent key environmental drivers underlying the observed geographic differences [141, 142].

Scientific investigations have demonstrated that high-fat and high-sucrose diets accelerate the development of PCOS phenotypes in mouse models by disrupting estrous cycles, impairing folliculogenesis, and altering serum testosterone and luteinizing hormone levels [143]. Similarly, prenatally androgenized PCOS mouse models exhibit ovarian-independent uterine dysfunction and placental inflammation, conditions that are further exacerbated under high-fat diet exposure [144]. These findings from animal studies highlight the detrimental role of unhealthy dietary patterns in PCOS progression. Consequently, there is growing interest in exploring healthy dietary approaches for the clinical management of PCOS. The Mediterranean diet (MedDiet), renowned for its anti-inflammatory, antineoplastic, anti-obesogenic, and antioxidant properties, is considered a gold-standard dietary model in preventive medicine [145, 146]. This eating pattern is characterized by regular consumption of unsaturated fats, dietary fiber, low-glycemic-index carbohydrates, antioxidants, vitamins, and moderate amounts of both animal and plant proteins. Studies have demonstrated that the MedDiet confers protection against obesity, cardiovascular disease, type 2 diabetes, and non-alcoholic fatty liver disease-benefits mediated through reduced inflammation and oxidative stress, improved lipid profiles, enhanced insulin sensitivity, better endothelial function, and antiatherogenic and antithrombotic effects [147–149]. Given the close associations between PCOS, obesity, chronic low-grade inflammation, and IR, the MedDiet emerges as a leading non-pharmacological strategy for PCOS management. Central to its benefits are plant polyphenols, derived from vegetables, fruits, legumes, whole grains, nuts, and seeds, which have been shown to attenuate inflammatory pathways, improve insulin sensitivity, and mitigate compensatory hyperinsulinemia in women with PCOS [150, 151]. Clinical investigations reinforce these mechanistic insights. An evaluation of 112 women with PCOS and matched healthy controls revealed that despite comparable energy intake, PCOS patients consumed fewer complex carbohydrates, fiber, monounsaturated fatty acids, and *n*-3 polyunsaturated fatty acids (PUFAs), and more simple carbohydrates, saturated fats, and *n*-6 PUFAs-suggesting an inverse correlation between adherence to a Mediterranean-style diet and PCOS severity [118]. Additionally, women with PCOS exhibited weight loss, underscoring the value of nutritional and body composition assessment in clinical management. Further supporting the therapeutic role of MedDiet, a 12-week randomized controlled trial conducted by Mei et al. demonstrated that a Mediterranean-based low-carbohydrate regimen outperformed a low-fat diet in restoring menstrual cyclicity, improving anthropometric measures, correcting reproductive endocrine abnormalities, enhancing insulin sensitivity, and optimizing lipid profiles in overweight/obese PCOS patients [119]. Similarly, Kowalczyk et al. reported that reducing intake of sweets, high-fat red meat, and alcohol significantly decreased total testosterone levels in women with PCOS [120]. Regarding dietary interventions, very-low-calorie diets appear more promising than moderate energy deficit diets for PCOS management. The very-low-calorie diet group showed significant improvements in sex hormone-binding globulin levels, fasting blood glucose, and waist-to-hip ratio, changes not observed in the moderate energy restriction group [121]. A meta-analysis also indicated that both ketogenic and low-calorie ketogenic diets were associated with reductions in body mass index and body fat percentage, along with improvements in glycemic control-specifically lowering fasting glucose and Homeostatic Model Assessment of IR values-compared to conventional low-calorie diets [122]. In overweight women with PCOS, ketogenic diets significantly reduced body mass index, fat body mass, triglycerides, total cholesterol, and low-density lipoprotein levels, while increasing high-density lipoprotein [123]. These findings were corroborated by Cincione and Sharifi et al., who reported that ketogenic diets, Portfolio Moderate-carbohydrate diets, and moderate low-calorie Mediterranean diets all significantly improved pathological features in overweight and obese PCOS patients [124, 125]. Regarding reproductive function, ketogenic diets restored normal menstrual cycles in all PCOS patients [126]. Rossetti et al. similarly reported that gradual carbohydrate reintroduction following ketogenic diet intervention successfully restored menstrual cyclicity in women with PCOS and oligomenorrhea [127]. Furthermore, β-hydroxybutyrate, a key metabolite of ketogenic diets, substantially reduced androgen and glucose levels in PCOS women [128]. Consequently, ketogenic diets may represent a promising option for selected overweight or insulin-resistant PCOS patients, although their long-term safety and adherence require further evaluation [152–154]. Likewise, high-fiber dietary interventions have been shown to significantly improve fasting glucose, IR, triglyceride, and low-density lipoprotein-cholesterol levels, while increasing HDL-cholesterol in women with PCOS [129]. Similarly, high-protein, low-calorie diets showed beneficial effects, with both low-calorie high-protein diets and conventional isocaloric diets significantly improving PCOS phenotypes, though the high-protein approach proved superior for reducing body fat percentage [130]. Beyond standalone dietary interventions, combined dietary and pharmacological approaches have shown enhanced therapeutic effects. The combination of high-fiber diet and acarbose produced stronger improvements in PCOS phenotypes and gut microbiota diversity compared to high-fiber diet alone [131]. Similarly, dulaglutide combined with calorie-restricted diet demonstrated superior efficacy versus calorie restriction alone [132].

Despite the compelling evidence supporting dietary interventions for PCOS, their successful clinical implementation requires careful consideration of several practical factors. Intervention feasibility and patient adherence remain paramount challenges, as restrictive diets such as ketogenic and very-low-calorie approaches, while efficacious in the short term, are difficult to sustain and may be inaccessible to socioeconomically disadvantaged populations. Patient stratification is essential given the heterogeneity of PCOS: obese, insulin-resistant individuals may benefit more from ketogenic diets, whereas lean patients might respond better to Mediterranean or high-fiber patterns, and cultural adaptation of dietary recommendations is necessary across diverse ethnic populations. Safety considerations mandate clinical oversight, as very-low-calorie and ketogenic diets carry risks of micronutrient deficiencies, dyslipidemia, and excessive lean mass loss, while high-protein diets may impose renal workload in susceptible individuals; furthermore, the safety of restrictive diets during pregnancy remains inadequately characterized. Finally, dietary interventions should be integrated with conventional therapies rather than viewed as alternatives, as combined approaches demonstrate superior efficacy, though optimal sequencing and potential medication-sparing effects require further investigation. Advancing the field requires future research to focus on three key areas: optimizing patient stratification, improving long-term dietary adherence, and establishing comprehensive safety guidelines to unlock the full therapeutic potential of dietary interventions for PCOS.

### Application of probiotics, prebiotics, and synbiotics

Microbiota-targeted therapies have been increasingly applied in the management of PCOS. Clinical studies indicate that probiotic supplementation can yield beneficial metabolic effects. For instance, a study involving 72 women with PCOS showed that daily administration of Familact probiotic capsules-containing *Lactobacillus*, *Bifidobacterium*, and *Streptococcus* strains-significantly improved serum insulin levels [133]. Similarly, Darvishi et al. reported that a multi-strain probiotic formulation comprising *Lactobacillus*, *Bifidobacterium*, and *Streptococcus* led to a notable increase in high-density lipoprotein (HDL) levels, although no significant differences were observed in total cholesterol, triglycerides, or low-density lipoprotein [134]. Regarding prebiotic interventions, Shamasbi et al. demonstrated that daily supplementation with 20 mg of resistant dextrin for three months significantly increased HDL concentrations and ameliorated menstrual cycle regularity and hirsutism in PCOS patients [135]. Beyond standalone probiotics and prebiotics, synbiotic therapies-combining probiotics and prebiotics-have also been widely investigated. In a trial involving 92 PCOS participants who received an 8-week intervention, three groups were compared: one receiving pomegranate juice (2 L/week), another a synbiotic beverage (2 L/week, containing water, 20 g inulin, 2 × 10^8^ CFU/g *Lactobacillus*, and pomegranate flavoring), and a third group receiving synbiotic pomegranate juice (2 L/week, enriched with inulin and *Lactobacillus*). The two synbiotic groups exhibited significantly enhanced insulin sensitivity and reduced testosterone levels [136]. Corroborating these findings, Karimi et al. observed marked clinical improvement in PCOS patients following a 12-week regimen of a synbiotic compound containing *Lactobacillus*, *Bifidobacterium*, *Streptococcus*, and inulin [137]. Likewise, supplementation with a synbiotic consisting of *Lactobacillus*, *Bifidobacterium*, and 800 mg of inulin significantly improved insulin sensitivity, reduced serum triglycerides, and attenuated the atherogenic index in women with PCOS [138]. However, a study by Arab et al. using a synbiotic preparation composed of *Lactobacillus*, *Lacticaseibacillus*, *Bifidobacterium*, *Streptococcus*, and 21 mg of inulin reported a significant increase in sex hormone-binding globulin levels, though no other significant hormonal or clinical improvements were noted [139]. The variability in synbiotic efficacy across clinical studies may be attributed to both formulation heterogeneity and patient population diversity. Key contributing factors encompass strain-specific effects of probiotic constituents, differential prebiotic dosing regimens, and inherent variations in host metabolic and microbiological profiles among PCOS cohorts. This collective evidence suggests that therapeutic outcomes are contingent upon precise formulation design and individualized intervention strategies. Collectively, these studies suggest that synbiotic interventions may offer superior therapeutic benefits compared to probiotic or prebiotic monotherapy in the management of PCOS. Notably, given the strain-specific and host-dependent effects of probiotics and synbiotics, future trials should select strains based on defined functional targets, such as SCFA production, bile acid regulation, gut barrier protection, or anti-inflammatory activity.

### Exploration of fecal microbiota transplantation (FMT)

FMT, a therapeutic strategy aimed at comprehensively reconstructing the gut microbial ecosystem, has demonstrated remarkable efficacy in conditions such as recurrent *Clostridium difficile* infection. However, its application in PCOS remains in a very early exploratory stage [155]. The core mechanism of FMT involves the introduction of a complete microbial community from a healthy donor to restore the diversity and functional stability of the host’s gut microbiota. Notably, in the field of metabolic diseases, FMT has been experimentally used in the management of type 2 diabetes, obesity, and non-alcoholic fatty liver disease [156, 157]. These findings lay a theoretical foundation for the potential application of FMT in PCOS, which similarly features IR and chronic inflammation as central pathological elements. These findings from metabolic diseases provide a solid foundation for envisioning FMT as a potential future intervention strategy for PCOS. The central premise lies in utilizing FMT to correct the characteristic gut dysbiosis in PCOS, which may indirectly modulate key pathways closely associated with the disease. This includes restoring normal bile acid metabolism to improve insulin signaling, increasing the production of beneficial metabolites such as SCFAs to enhance gut barrier integrity and reduce inflammation, ultimately potentially creating a favorable systemic and local microenvironment for alleviating hyperandrogenemia and ovarian dysfunction. Critical next steps will involve validating the feasibility of this approach through rigorous preclinical studies, followed by initiating early-phase clinical trials to evaluate the actual interventional effects of FMT on core PCOS symptoms.

Despite its theoretical promise for fundamentally reconstructing the gut ecosystem in PCOS, the translation of FMT into clinical practice confronts substantial practical challenges that warrant careful consideration. Foremost among these is intervention feasibility, as FMT necessitates rigorous donor screening, standardized fecal material preparation, and professional medical oversight. Patient selection represents another critical consideration. Given the marked heterogeneity of PCOS, not all individuals are suitable candidates for FMT. Identifying subpopulations most likely to derive meaningful benefit constitutes an essential step toward enhancing the clinical relevance and therapeutic precision of this approach. Patients with demonstrable severe gut dysbiosis, particularly those characterized by reduced microbial diversity and depletion of SCFA-producing bacteria, may represent the most appropriate target population. Additionally, PCOS patients with prominent inflammatory phenotypes or those who have failed to respond adequately to conventional dietary and pharmacological interventions warrant consideration as potential candidates for this emerging therapeutic strategy. Safety considerations arguably pose the most significant barrier to FMT implementation in PCOS. Despite rigorous donor screening protocols, potential risks include transmission of undetected pathogens, induction of autoimmune or inflammatory conditions through inappropriate immune modulation, and unintended metabolic disturbances resulting from the introduction of exogenous microbial communities. Furthermore, there remains a lack of consensus regarding optimal donor selection criteria. Given these considerations, future research must proceed with appropriate caution. Priorities include rigorous preclinical evaluation, carefully designed early-phase clinical trials incorporating comprehensive safety monitoring, and parallel development of standardized regulatory frameworks. Only through such methodical progression can FMT be responsibly considered for clinical application in PCOS.

### Translational framework for microbiota-based interventions in PCOS

Although microbiota-based interventions have shown therapeutic potential in PCOS, their clinical application should be guided by a stratified framework rather than applied uniformly to all patients. Given the heterogeneity of PCOS, a practical framework should begin with baseline clinical phenotyping, including BMI, waist circumference, insulin resistance, androgen excess, menstrual and ovulatory status, inflammatory markers, medication exposure, and dietary habits. These clinical features should then be integrated with microbiota and metabolite profiles, such as microbial diversity, SCFA-producing taxa, fecal or serum SCFA levels, bile acid metabolism, and potentially pro-inflammatory microbial signatures. This combined assessment may help identify patient subgroups that are more likely to benefit from specific microbiota-targeted interventions.

Intervention selection should be guided by both clinical phenotype and microbial characteristics. For obese or insulin-resistant PCOS patients, Mediterranean-style diets, low-carbohydrate dietary patterns, dietary fiber supplementation, probiotics, synbiotics, or metformin-associated microbiota modulation may be considered to improve insulin sensitivity and metabolic dysfunction. Patients with reduced microbial diversity, depletion of SCFA-producing genera such as *Faecalibacterium* and *Roseburia*, or reduced fecal SCFA levels may be more suitable for butyrate-oriented interventions [158, 159]. For patients with prominent inflammatory features or suspected intestinal barrier dysfunction, strategies aimed at restoring microbial homeostasis, enhancing gut barrier integrity, and reducing chronic low-grade inflammation may be prioritized. Medication history should also be considered, as metformin and hormonal therapies may reshape gut microbiota and influence treatment response. In contrast, FMT should not currently be regarded as a routine clinical option for PCOS, but rather as an exploratory approach for carefully selected patients with severe dysbiosis, low microbial diversity, depletion of beneficial metabolite-producing bacteria, or poor responses to conventional dietary and pharmacological therapies. Clinical efficacy should be evaluated using multidimensional endpoints rather than relying solely on changes in microbiota composition. Reproductive outcomes, including menstrual cyclicity, ovulation, androgen levels, and ovarian morphology, should be assessed together with metabolic indicators such as BMI, waist circumference, fasting glucose, fasting insulin, HOMA-IR, lipid profiles, and inflammatory markers. Microbiota-related endpoints, including microbial diversity, SCFA-producing taxa, fecal or serum SCFA levels, bile acid profiles, and other microbial metabolites, may help determine whether clinical improvement is accompanied by functional remodeling of the gut ecosystem. This integrated assessment would provide a more comprehensive evaluation of both therapeutic efficacy and microbiota-mediated mechanisms.

Based on this translational framework, future clinical trials should formulate specific and testable hypotheses according to both clinical phenotypes and microbiota-related features. For example, a stratified trial could test whether insulin-resistant PCOS patients with reduced SCFA-producing bacteria achieve greater improvements in HOMA-IR, androgen levels, menstrual cyclicity, and inflammatory markers after high-fiber, prebiotic, or synbiotic interventions than unstratified PCOS patients receiving the same intervention. Another testable hypothesis is that baseline depletion of butyrate-producing genera, such as *Faecalibacterium* and *Roseburia*, or reduced fecal SCFA concentrations may predict a favorable response to butyrate-oriented microbiota interventions. In metformin-treated PCOS patients, longitudinal studies could examine whether improvements in insulin sensitivity and endocrine parameters are partly mediated by gut microbiota remodeling and associated changes in bile acid or SCFA metabolism. For FMT, early-phase controlled trials should test whether carefully selected patients with severe dysbiosis and poor responses to conventional therapy derive greater metabolic or reproductive benefit than unselected patients. These hypothesis-driven designs would help identify PCOS subgroups most likely to benefit from microbiota-targeted interventions, define microbial or metabolic biomarkers of treatment response, and clarify whether gut microbiota remodeling functionally contributes to clinical improvement.

## Research challenges and future prospects

Current research on the association between PCOS and the gut microbiota still faces several limitations. Firstly, there is substantial heterogeneity in study design: most human studies are characterized by small sample sizes and cross-sectional designs, lacking unified methodologies for the gut microbiota detection and standardized stratification criteria for PCOS subtypes. This makes it challenging to clarify the causal relationship between the gut microbiota and PCOS. Secondly, the depth of mechanistic research remains insufficient: although it has been confirmed that the gut microbiota influences PCOS via inflammatory and metabolic pathways, the specific targets of individual bacterial strains and the precise interaction mechanisms between microbial metabolites and host endocrine pathways have not yet been fully elucidated. Thirdly, clinical translation bottlenecks exist: critical information such as strain selection, dosage standards, and long-term safety data for microbiota-targeted interventions is lacking. Additionally, differences in the gut microbiota composition among distinct PCOS populations (adolescents and obese patients) have not been fully considered, hindering the development of personalized intervention strategies.

In response to these limitations, emerging research efforts are actively pursuing innovative approaches to overcome these bottlenecks. A particularly promising strategy involves repurposing existing drugs with well-established safety profiles or leveraging plant-derived bioactive compounds for PCOS treatment, while simultaneously elucidating the underlying mechanisms involving gut microbiota. This dual approach not only accelerates the timeline for clinical translation but also provides deeper mechanistic insights that may reveal novel therapeutic targets. Yating et al. demonstrated that metformin, a first-line medication for type 2 diabetes with a long-standing safety record, improves gut microbiota composition and increases serum indole-3-acetic acid (I3A) levels, thereby activating AMPK signaling to ameliorate PCOS phenotypes [117]. This finding not only repurposes an existing safe drug for PCOS management but also reveals a gut microbiota-dependent mechanism underlying metformin’s therapeutic effects. Lulu et al. reported that supplementation with inulin, a plant-derived prebiotic fiber, increases the abundance of *Bifidobacterium* and other SCFA-producing bacteria, exerting beneficial regulatory effects on metabolic disturbances and ovarian dysfunction in PCOS [81]. As a naturally occurring dietary fiber already widely consumed, inulin represents a readily translatable intervention with minimal safety concerns. Yanxiang et al. discovered that naringenin, a flavonoid compound abundant in citrus fruits, exhibits therapeutic potential in PCOS through modulation of the gut microbiota and the SIRT1/PGC-1α signaling pathway [160]. These investigations collectively provide novel insights into the clinical application of gut microbiota modulation for PCOS management. Greater attention should be directed toward existing drugs and plant-derived monomers with therapeutic potential for PCOS. On one hand, their safety profiles have been sufficiently validated through years of clinical use or dietary consumption, conferring stronger translational significance and potentially bypassing the lengthy safety evaluation required for novel compounds. On the other hand, the relatively defined composition and known pharmacokinetic properties of these monomers facilitate deeper mechanistic exploration, enabling researchers to dissect the precise pathways through which they modulate gut microbiota and host physiology. By elucidating these mechanisms, we may identify additional molecular targets, ultimately paving the way for more precise and effective therapeutic strategies for PCOS.

While significant progress has been made in elucidating the pathogenesis of PCOS, the interplay between the gut microbiota and its metabolites with the immune system, as well as their impact on key PCOS symptoms, has been partially understood [161]. The influence of the gut‑vagina axis on PCOS, along with alterations in both vaginal and gut microbial communities, has also been explored [162]. Furthermore, the emergence of personalized intervention strategies tailored to different BMI subtypes reflects the trend toward increasingly refined management of PCOS as research advances [163]. This review has synthesized the underlying mechanisms linking gut microbiota to PCOS and, by incorporating the latest literature, further clarified the clinical applications of microbiota‑based regulation in PCOS. However, despite these advances, a substantial gap remains between current research findings and their translation into practical, clinically implementable strategies, necessitating continued breakthroughs in this field.

Future research can achieve breakthroughs in three key areas. In terms of underlying mechanisms, techniques such as genetic editing bacteria strain and single-cell sequencing should be employed to dissect the molecular pathways through which specific microbiota regulate ovarian function and insulin sensitivity, thereby clarifying the precise “microbiota-metabolite-host” interaction network. In clinical research, multi-center, large-sample longitudinal cohort studies are needed to establish classification standards for gut microbiota profiles in PCOS. Integrating metabolomics and transcriptomics data will further enable the construction of disease prediction models. In clinical translation, efforts should focus on developing PCOS-specific probiotic formulations, optimizing donor selection and transplantation protocols for FMT, and exploring the synergistic effects of lifestyle factors with microbiota interventions. Ultimately, these advances will facilitate the gut microbiota-based precise diagnosis and stratified treatment of PCOS, providing novel strategies to improve patients’ reproductive and metabolic outcomes.

## Data Availability

No datasets were generated or analyzed during the current study.
